# The Structural and Functional Organization of the Podocyte Filtration Slits Is Regulated by Tjp1/ZO-1

**DOI:** 10.1371/journal.pone.0106621

**Published:** 2014-09-03

**Authors:** Masahiko Itoh, Kazuhiko Nakadate, Yasuhiro Horibata, Taiji Matsusaka, Jianliang Xu, Walter Hunziker, Hiroyuki Sugimoto

**Affiliations:** 1 Department of Biochemistry, School of Medicine, Dokkyo Medical University, Mibu-machi, Shimotsuga-gun, Tochigi, Japan; 2 Department of Basic Biology, Educational and Research Center for Pharmacy, Meiji Pharmaceutical University, Kiyose, Tokyo, Japan; 3 Department of Internal Medicine, Tokai University School of Medicine, Isehara, Kanagawa, Japan; 4 Epithelial Cell Biology Laboratory, Institute of Molecular and Cell Biology (IMCB), Singapore, Singapore; University of North Carolina at Chapel Hill, United States of America

## Abstract

Blood filtration in the kidney glomerulus is essential for physiological homeostasis. The filtration apparatus of the kidney glomerulus is composed of three distinct components: the fenestrated endothelial cells, the glomerular basement membrane, and interdigitating foot processes of podocytes that form the slit diaphragm. Recent studies have demonstrated that podocytes play a crucial role in blood filtration and in the pathogenesis of proteinuria and glomerular sclerosis; however, the molecular mechanisms that organize the podocyte filtration barrier are not fully understood. In this study, we suggest that tight junction protein 1 (Tjp1 or ZO-1), which is encoded by *Tjp1* gene, plays an essential role in establishing the podocyte filtration barrier. The podocyte-specific deletion of *Tjp1* down-regulated the expression of podocyte membrane proteins, impaired the interdigitation of the foot processes and the formation of the slit diaphragm, resulting in glomerular dysfunction. We found the possibility that podocyte filtration barrier requires the integration of two independent units, the pre-existing epithelial junction components and the newly synthesized podocyte-specific components, at the final stage in glomerular morphogenesis, for which *Tjp1* is indispensable. Together with previous findings that *Tjp1* expression was decreased in glomerular diseases in human and animal models, our results indicate that the suppression of *Tjp1* could directly aggravate glomerular disorders, highlights *Tjp1* as a potential therapeutic target.

## Introduction

Renal glomerulus is an indispensable system for ultrafiltration of the blood and ensures that essential plasma proteins are retained. Glomerular filtration apparatus is composed of three distinct components: the fenestrated endothelial cells, the glomerular basement membrane (GBM), and podocytes. Glomerular dysfunction is marked by the loss of protein in the urine or proteinuria, which leads to end-stage renal disease due to sclerosis of the glomerulus [1]. Recent studies have clarified that the loss of function of the components of the glomerular podocyte has been implicated in progressive renal diseases such as diabetic nephropathy and focal segmental glomerulosclerosis (FSGS) [2]. *NPHS1* and *NPHS2*, the responsible genes for Finnish-type congenital nephrotic syndrome and autosomal recessive steroid-resistant nephrotic syndrome, respectively, encode nephrin and podocin, both of which are expressed in podocytes [3], [4]. Mutations in other podocyte-expressed molecules such as α-actinin-4 and CD2AP have also been associated with congenital nephrotic syndrome [5], [6]. The mice that are deficient for these molecules have exhibited renal diseases; while the age of onset may be varied, indicating that the molecules expressed in the podocytes regulate glomerular functions [7]-[10].

Glomerular podocytes are highly differentiated epithelial cells that extend numerous actin-rich projections known as foot processes, which interdigitate and cover the capillary walls of the glomerulus [11]. At the site of interdigitation, a specialized intercellular junction, the slit diaphragm, forms to function as the final sieve of the glomerular filter [12]. Previous studies have indicated that these slit diaphragms are modified tight junctions or adherens junctions, both of which play crucial roles in the epithelial tissue architecture as apical junctional complexes [13]–[15]. During the podocyte differentiation, the structures of the apical junctional complexes disappear and are replaced by the slit diaphragms; several components of the apical junctional complexes, including P-cadherin, β-catenin, and tight junction protein 1 (Tjp1 or ZO-1) are then localized at the slit diaphragm and form molecular complexes with the actin filaments and podocyte-specific proteins [16], [17]. In addition, the *Drosophila* orthologue of Tjp1 as well as nephrin and podocin are expressed in nephrocytes, which have structural and functional similarities with podocytes [18], suggesting the possibility that the essential elements for the filtration system have been molecularly and architecturally conserved during evolution.

The several previous studies have indicated that tight junctions and *Tjp1* are implicated in glomerular disorders. Tight junctions reappear between adjoining foot processes during certain proteinuria-associated glomerular diseases and animal models [19], [20]. *Tjp1* expression is significantly decreased in the glomeruli of human diabetic kidneys [20]–[22]. Furthermore, animal models of both type 1 and 2 diabetes including *db/db* mice and pharmacologically-induced diabetic rats have exhibited the reduction and redistribution of Tjp1 in glomerular podocytes [20]–[22].

These observations suggest the possibility that the glomerular filtration system is affected by *Tjp1* under physiological and pathological conditions; however, its direct and functional relevance remains unclear. In the current study, to improve our understanding of how the glomerular filtration system is regulated, we specifically inactivated *Tjp1* in glomerular podocytes in mice and found that *Tjp1* plays an essential role in the formation and maintenance of the podocyte filtration barrier.

## Results

### Podocyte-specific deletion of *Tjp1* leads to global glomerulosclerosis

**Figure 1:**
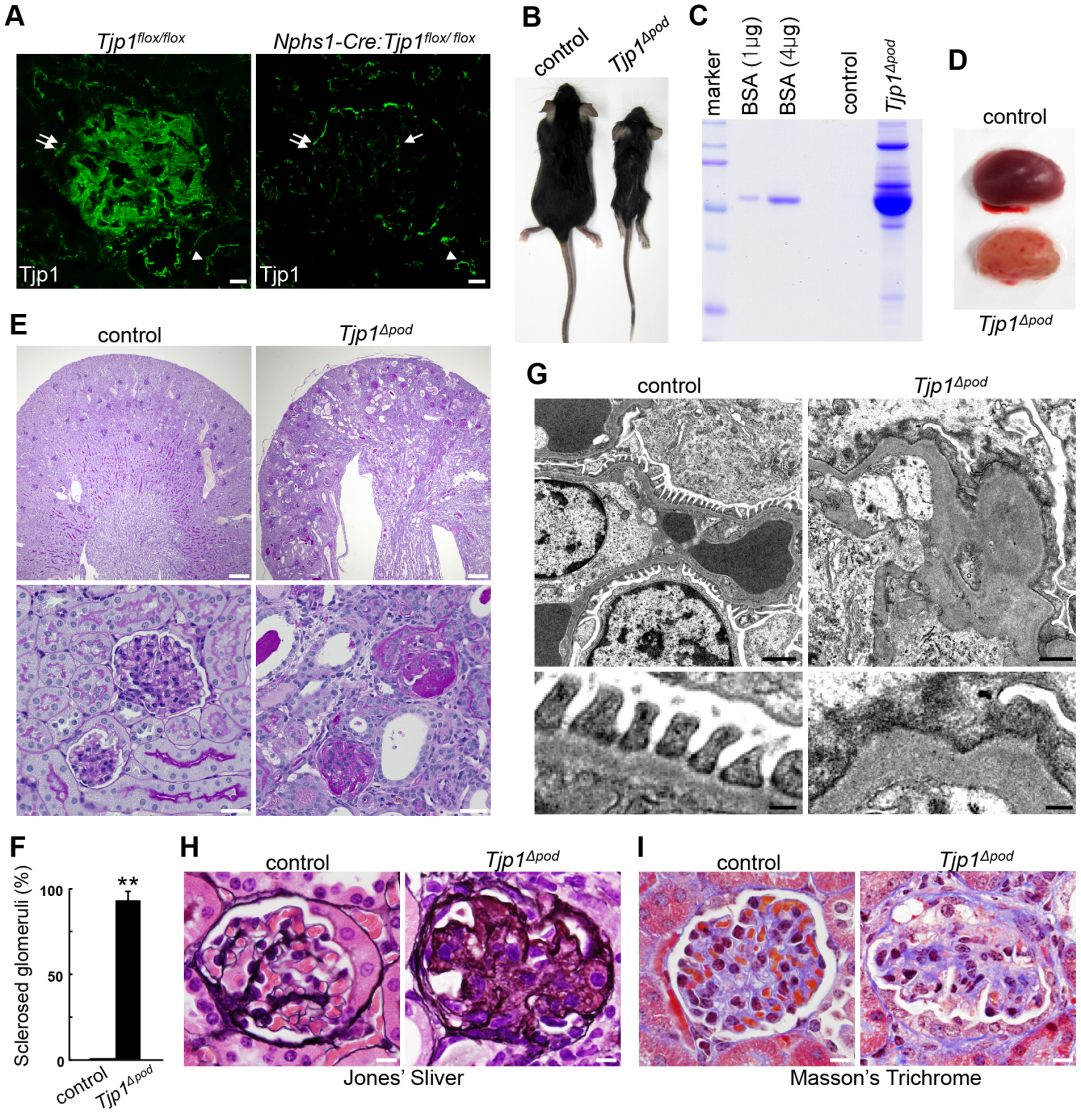
Podocyte-specific deletion of *Tjp1* results in glomerulosclerosis. (A) Kidney sections from *Tjp1^flox/flox^* and *Nphs1-Cre: Tjp1^flox/flox^* mice were examined with the antibody against Tjp1. Tjp1 was specifically eliminated from podocytes, while it was detected in endothelial cells in the glomerulus in the *Nphs1-Cre: Tjp1^flox/flox^* mice (arrow). The expression of Tjp1 in the Bowman's capsule epithelial cells (double arrows) and in the renal tubules (arrowheads) was also unaffected. See also Fig. S2. Scale bars, 10 µm. (B–I) Analyses of the control (*Tjp1^flox/flox^*) and *Tjp1^△pod^* (*Nphs1-Cre: Tjp1^flox/flox^*) mice at 6 weeks of age. The *Tjp1^△pod^* mice exhibited significant growth retardation (B) and developed massive proteinuria (C). (D) Gross appearance of the kidneys from the control and *Tjp1^△pod^* mice at 6 weeks of age. (E) Histological analyses with periodic acid-Schiff (PAS) staining displayed disorder in the tissue architecture of the *Tjp1^△pod^* mice kidney (top panels). Severe glomerulosclerosis and proteinaceous casts in the dilated renal tubules were observed in the *Tjp1^△pod^* mice, but not in control mice (bottom panels). Scale bars, 200 µm (top panels), 20 µm (bottom panels). (F) The number of sclerosed glomeruli was expressed as a percentage of the total number of glomeruli (mean ± SEM of n = 3, **p<0.001). (G) Transmission electron micrographs showing the loss of slit diaphragms, destruction of foot processes, and aberrant glomerular basement membranes in the *Tjp1^△pod^* mice. Scale bars, 2 µm (top panels), 0.5 µm (bottom panels). (H and I) Histopathological analyses by Jones' silver (H) and Masson's trichrome (I) stain. Global sclerosis with extensive deposits of basement membrane components were observed with both stains. Scale bars, 10 µm.

To delete *Tjp1* specifically from podocytes, we utilized newly generated *Tjp1^flox/flox^* mice (Fig. S1) and *Nphs1-Cre* transgenic mice, which drive Cre recombinase expression in podocytes under nephrin promoter [23]. First, we verified the podocytes-specific *Tjp1* inactivation by staining kidney sections from *Tjp1^flox/flox^* mice and *Nphs1-Cre: Tjp1^flox/flox^* mice (Fig. 1A and Fig. S2). In the *Nphs1-Cre: Tjp1^flox/flox^* mice, the signal for Tjp1 was absent from glomerular podocytes (Fig. 1A and Fig. S2A), whereas a positive signal was observed in the endothelial cells (Fig. 1A, arrow and Fig. S2B), in the Bowman's capsule epithelial cells (Fig. 1A, double arrows and Fig. S2C), and the cell–cell junctions of the renal tubules (Fig. 1A, arrowheads). The reduction of Tjp1 protein was also confirmed by Western blotting analysis for the glomerular lysates (Fig. S2D). These data indicated that *Tjp1* was eliminated specifically from the podocytes. In addition, we examined the expression of the Tjp family members, Tjp2 and Tjp3 in the glomerulus [24]. Immunofluorescence analyses revealed that the intensity and staining pattern of either Tjp2 or Tjp3 was not altered in the *Nphs1-Cre: Tjp1^flox/flox^* mice (*Tjp1^△pod^*) compared with the pattern in the *Tjp1^flox/flox^* mice (control) (Fig. S3A and B), suggesting that these molecules could not compensate for the loss of *Tjp1* in the podocytes.

The *Tjp1^△pod^* mice were born according to Mendelian rules, but exhibited significant growth retardation (Fig. 1B) and severe proteinuria (Fig. 1C). Kidneys from the *Tjp1^△pod^* mice at 6 weeks of age were pale and had a more granular surface compared with the kidneys from the control mice (Fig. 1D). The histological analyses of these kidneys demonstrated that the renal pelvis was enlarged with atrophic renal papilla (Fig. 1E, top panels); prominent glomerulosclerosis and dilated tubuli containing proteinaceous casts were detected in the *Tjp1^△pod^* mice as well (Fig. 1E, bottom panels). We found more than 90% of glomeruli showed sclerosis in the *Tjp1^△pod^* mice (Fig. 1F). Transmission electron microscopy revealed the loss of the slit diaphragm, the disruption of foot processes, and the aberrantly thickened, tortuous GBM in the *Tjp1^△pod^* mice at 6 weeks of age (Fig. 1G).

To investigate whether the kidney disorder caused by the podocyte-specific *Tjp1* inactivation had a similarity with human glomerular diseases, we performed pathological analyses that have been commonly utilized in the diagnosis of human renal disease [25]. In the *Tjp1^△pod^* mice, Jones' silver and Masson's trichrome staining revealed extensive deposits of the basement membrane components. (Fig. 1H and I). These pathological characteristics were comparable to those in patients with global glomerulosclerosis.

Taken together, these data provided evidence that *Tjp1* is indispensable for the maintenance of glomerular structure and function and that the elimination of *Tjp1* leads to global glomerulosclerosis.

### *Tjp1* is required for the establishment of the glomerulus in immature mice

In mice, the renal glomerulus is fully established at 2–3 weeks after birth [26]. To define the effect of *Tjp1* deficiency on the establishment of the renal glomerulus, we performed retrospective analyses. The histology of the *Tjp1^△pod^* mice at 4 weeks of age demonstrated a severe sclerotic glomeruli (Fig. S3C). A milder disorder that was still evident histologically was observed in the *Tjp1^△pod^* mice at 2 weeks of age (Fig. S3D). At the ultrastructural level, we found effacement of the foot processes and slit diaphragm impairment in the *Tjp1^△pod^* mice, whereas the control mice displayed an intact arrangement of foot processes with preserved filtration slits at 2 weeks of age (Fig. S3E). We also noticed that the GBM is mostly well-organized with the clear three layers in the *Tjp1^△pod^* mice at 2 weeks of age (Fig. S3E), while the organization of GBM is severely impaired at 6 weeks of age (Fig. 1G), suggesting that the loss of slit diaphragms and foot process effacement precede the disorganization of the basement membrane in this mutant mouse. These findings, together with the presence of severe proteinuria in the *Tjp1^△pod^* mice at 2 weeks of age (Fig. S3F), indicate that *Tjp1* is necessary for the structural and functional establishment of renal glomerulus in immature mice.

### The interdigitating foot process architecture is disrupted in *Tjp1^△pod^* mice

The interdigitating foot processes are a crucial portion of the glomerular filtration apparatus, and it has been demonstrated that the foot processes retract and are lost in the glomerular diseases that are typically associated with proteinuria [2]. In fact, mice that were missing the slit diaphragm components have been shown to lose their foot processes [9], [10], [27], [28]. To gain further insight into the effect of *Tjp1* deletion on the interdigitating foot process architecture, the kidneys from both the control and *Tjp1^△pod^* mice were analyzed by scanning electron microscopy (SEM).

**Figure 2:**
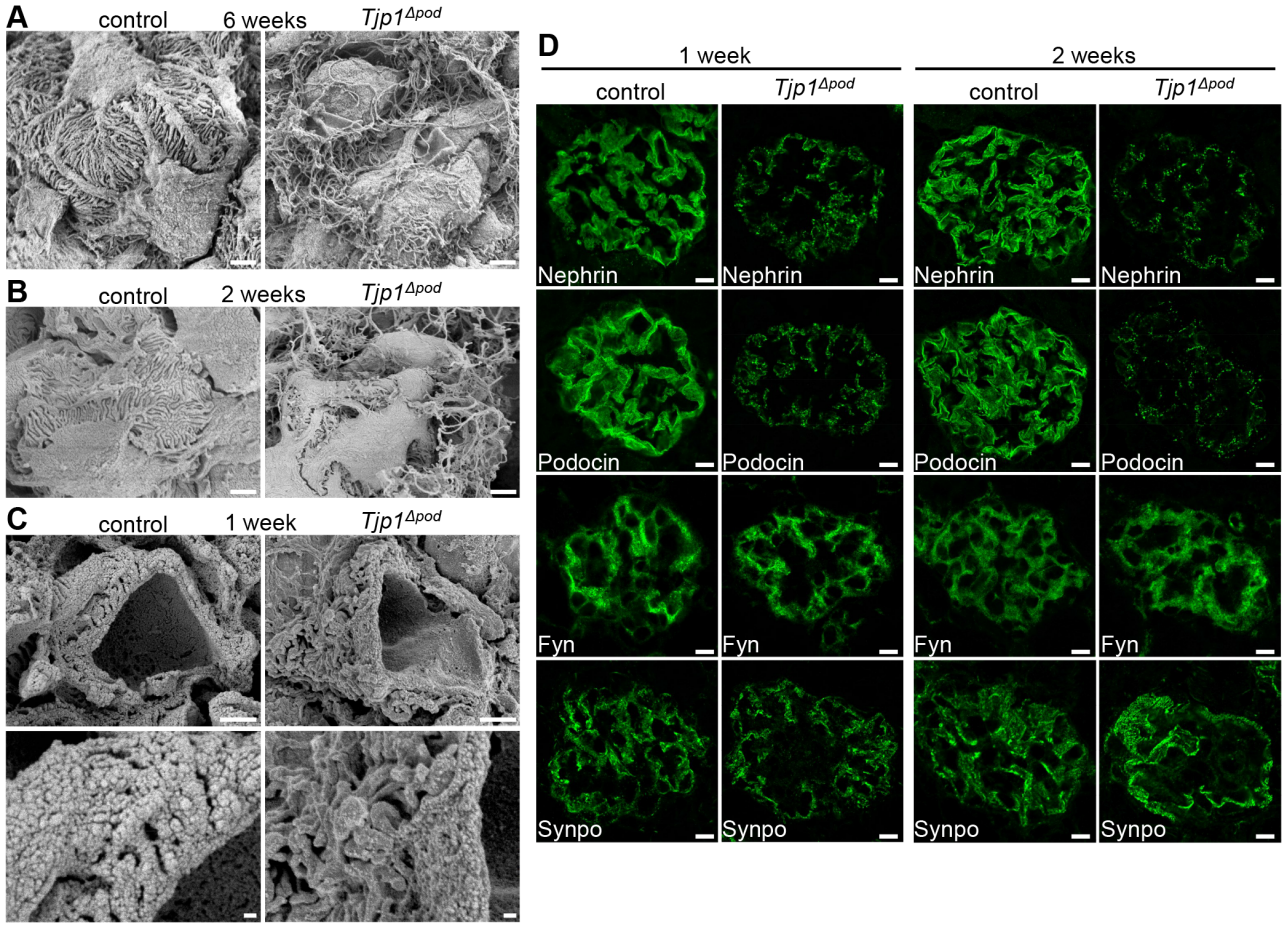
The compromised foot process interdigitation and aberrant distribution of the slit diaphragm components in *Tjp1^△pod^* mice. (A–C) SEM analyses of glomeruli from the control and *Tjp1^△pod^* mice. Foot processes were severely disorganized in the *Tjp1^△pod^* mice at 6 weeks of age (A). The loss of interdigitation and the impaired adhesion to the GBM of the podocyte foot processes was detected in the *Tjp1^△pod^* mice at 2 weeks of age (B). At an earlier time point, 1 week of age, the foot processes were attached to the GBM but the interdigitation was disorganized in the *Tjp1^△pod^* mice (C). Scale bars, 2 µm (A, B, C top panels), 0.5 µm (C bottom panels). (D) Immunofluorescence analyses of podocyte components: nephrin, podocin, Fyn, and synaptopodin (Synpo). The distribution of the slit diaphragm membrane proteins, nephrin and podocin, was fragmented and the signal intensity was reduced in the *Tjp1^△pod^* mice at 1 week and 2 weeks of age. The signal of the actin-binding protein Synpo was increased in the *Tjp1^△pod^* mice at 2 weeks of age. Fyn, a member of src family kinase, was not affected. See also Fig. S4A and C. Scale bars, 10 µm.

In the 6-week-old mice that did not have *Tjp1* in podocytes, the podocyte foot processes were spreading out randomly. The interdigitating architecture was completely disorganized and the adhesion of foot processes to the GBM appeared to be impaired (Fig. 2A). In addition, the primary processes were flatter compared with those in the control mice, suggesting the possibility that the organization of the actin filaments might be affected by the inactivation of *Tjp1*.

The disorder in the interdigitation and the adhesion to the GBM of foot processes became evident in the *Tjp1^△pod^* mice by 2 weeks of age (Fig. 2B). At 1 week of age, most of the foot processes appeared to adhere to the GBM, which surrounds the capillary walls, in both the control and *Tjp1^△pod^* mice (Fig. 2C, top panels), however, the adjoining foot processes in the *Tjp1^△pod^* mice did not interdigitate properly compared with those in the control mice. (Fig. 2C, bottom panels).

### The localization of the specific podocyte components is affected by *Tjp1* elimination

To explore the molecular mechanisms underlying these alterations, we examined the distribution of the podocyte components in the mice at 1 and 2 weeks of age. In the control mice, the slit diaphragm membrane proteins, nephrin and podocin, were distributed in a linear pattern presumably along the glomerular capillary wall; in contrast, those proteins appeared as discontinuous dots in the *Tjp1^△pod^* mice at both 1 and 2 weeks of age (Fig. 2D). The higher magnification images of podocin provided more conclusive evidence for the alteration of its spatial organization by *Tjp1* depletion in podocytes (Fig. S4A). In addition, immunoelectron microscopy showed that podocin was highly concentrated at the slit diaphragm in the control mice, whereas it was located near the GBM and around the disorganized cytoskeletal filaments in the *Tjp1^△pod^* mice (Fig. S4B).

The loss of *Tjp1* did not affect the localization of the src-family kinase Fyn, which binds to the cytoplasmic domain of nephrin [29]. On the other hand, an increased signal was observed for the actin-associated protein synaptopodin (Synpo) [30] in the *Tjp1^△pod^* mice compared with that in the control mice at 2 weeks of age; this finding was more evident in the higher magnification images (Fig. S4C).

### Loss of *Tjp1* alters the expression of podocyte components at post-transcriptional level

**Figure 3:**
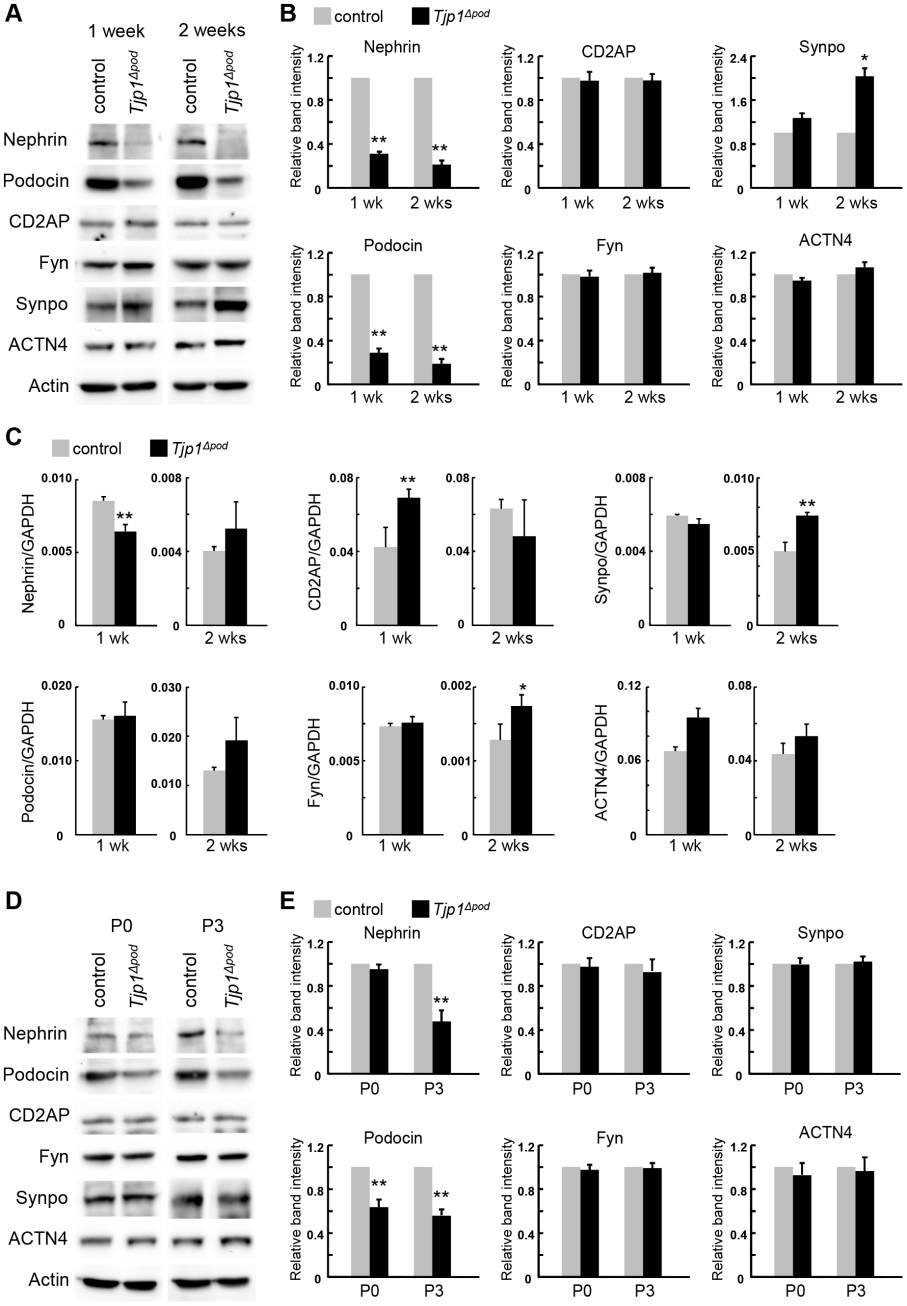
Post-transcriptional regulation alters the expression of Nephrin, Podocin, and Synpo in *Tjp1^△pod^* mice. (A and B) The glomerular lysates from the control and *Tjp1^△pod^* mice at 1 week and 2 weeks of age were analyzed by Western blotting with the antibodies against podocyte components (A). The results were quantified (B). Nephrin and podocin were significantly downregulated in the *Tjp1^△pod^* mice both at 1 week and 2 weeks of age. The considerable increase in the Synpo protein was observed in 2-week-old *Tjp1^△pod^* mice. The expression level of CD2AP, Fyn, and α-actinin4 (ACTN4) did not exhibit differences between the control and *Tjp1^△pod^* mice. (C) mRNA expression of podocyte components was determined by qPCR. The difference in the neprhin and podocin mRNA levels between the control and *Tjp1^△pod^* mice at 1 week and 2 weeks of age did not correlate with the reduction in the protein levels of those molecules. The mRNA level of most other molecules did not show these alterations, although some of them did exhibit upregulation without an increase in protein levels except for Synpo at 2 weeks of age. (D and E) The protein expression of the podocyte components in the developing glomerulus was examined by Western blotting. The kidney lysates from the control and *Tjp1^△pod^* mice at P0 and P3 were probed with antibodies against podocyte components (D). The quantification data indicated that podocin was downregulated at P0 and P3, while the reduction of nephrin protein was detected at P3. All data are represented as mean ± SEM of n = 3. *p<0.01, **p<0.001

Next, the protein levels of podocyte components were determined by Western blotting. In accordance with the immunostaining images, the protein expression of nephrin and podocin was significantly downregulated in the *Tjp1^△pod^* mice both at 1 and 2 weeks of age (Fig. 3A and B). The nephrin-binding cytoplasmic proteins, CD2AP and Fyn, did not exhibit differences in their expression between the control and *Tjp1^△pod^* mice, regardless of age. As for Synpo and α-actinin4 (ACTN4), which was another actin-associated protein in the podocytes [10], there was an almost two-fold increase detected specifically for Synpo in the *Tjp1^△pod^* mice at 2 weeks of age. While the reduction of nephrin or podocin has been frequently observed in human glomerular diseases and animal models, the increase in Synpo expression has not been previously described [12], [31].

To investigate whether the alteration in protein expression occurred at a transcriptional level, we performed quantitative PCR analysis (Fig. 3C). A substantial correlation between the protein and mRNA levels was not observed for nephrin and podocin. The Synpo mRNA level was increased in the *Tjp1^△pod^* mice at 2 weeks of age, which was consistent with the alteration in the protein level. However, it was not clear if the increase in the Synpo protein was a consequence of transcriptional upregulation because the mRNA levels of several of the other molecules also increased without exhibiting an increase in the protein level. Taken together, the alterations in the protein expression of nephrin, podocin, and Synpo were probably post-transcriptional events.

To determine when the reduction of nephrin and podocin protein started, we analyzed the kidney lysates from newborn mice (P0) and mice on postnatal day 3 (P3) (Fig. 3D and E). Both nephrin and podocin were specifically downregulated by P3. At P0, the nephrin protein level in the *Tjp1^△pod^* mice appeared to be comparable with the level in the control mice; however, podocin exhibited an obvious decrease in the *Tjp1^△pod^* mice (Fig. 3D and E). Therefore, the initial alteration in protein expression after the elimination of *Tjp1* in podocytes appeared to be the reduction of podocin.

### The stability of podocin is prolonged in the presence of Tjp1

**Figure 4:**
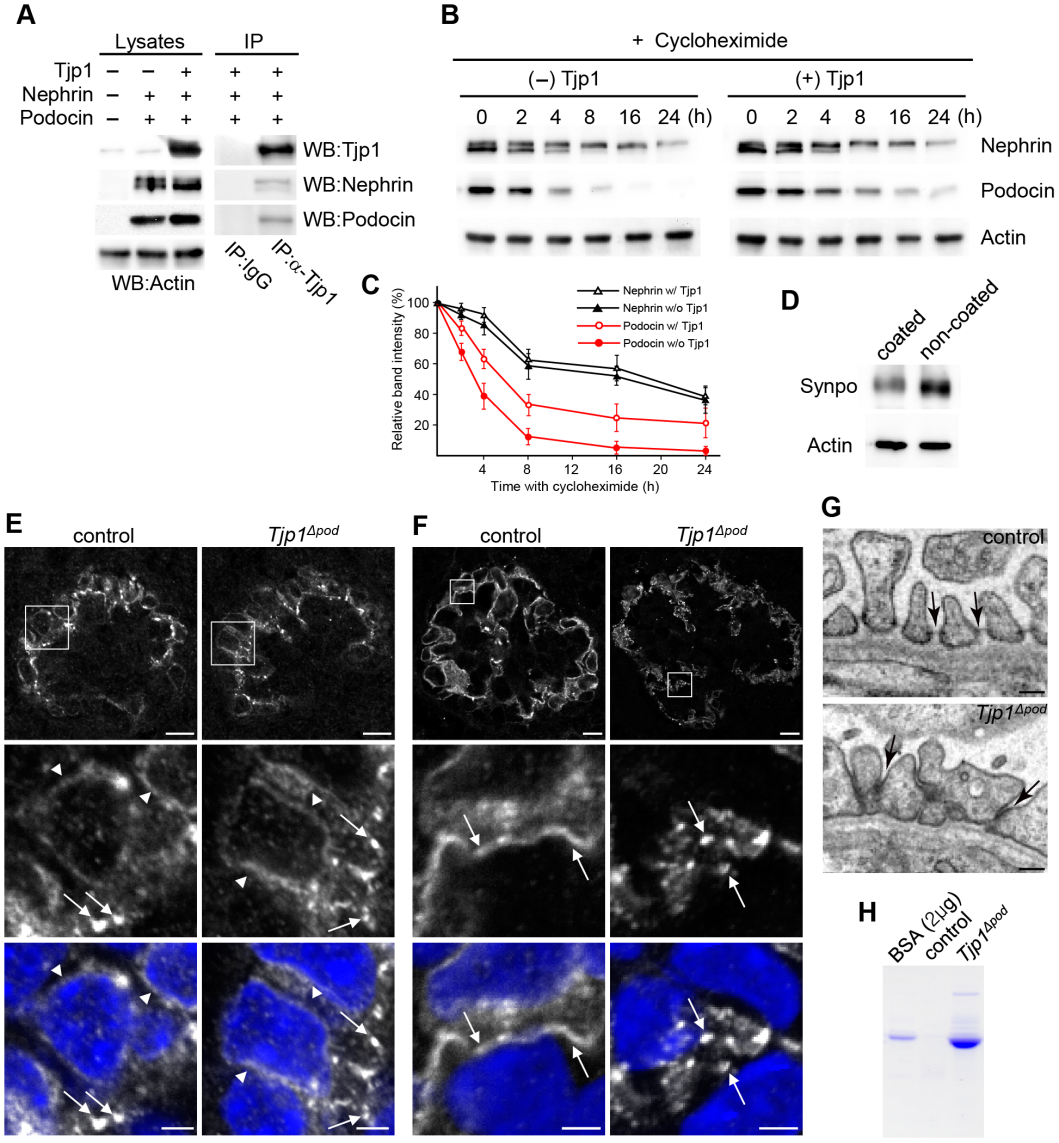
*Tjp1* affects the stability of podocin protein and the maturation of the slit diaphragm. (A) Nephrin and podocin were introduced into L cells with or without Tjp1. The expression of the transfected genes was examined by Western blotting of the cell lysastes and the protein complex formation was analyzed by the immunoprecipitation assay (IP) using anti-Tjp1 antibody or control IgG. (B and C) Cells stably expressing nephrin and podocin with or without Tjp1 were treated with 10 µM cycloheximide for an indicated amount of time. The protein levels of nephrin and podocin were analyzed by Western blotting (B) and the results were quantified (C). The relative expression of the band intensity at time 0 for each protein was defined as 100%. Data shows mean ± SEM of n = 3. (D) Mouse podocytes were seeded on the type-I collagen-coated dishes or non-coated petri dishes and cultured for 16 h, followed by the examination of the expression of Synpo by Western blotting. (E and F) Kidney sections from the control and *Tjp1^△pod^* mice at P0 were stained with anit- podocin antibody. (E) Before the maturing stage, podocin was detected around the nucleus (arrowheads), in addition, at the basolatral membrane domains as dots (arrows) both in the control and *Tjp1^△pod^* mice. (F) The distribution of podocin exhibited a linear pattern at the maturing stage in control mice (arrows in control), suggesting the formation of slit diaphragms. On the other hand, podocin was not integrated into a linear pattern in the *Tjp1^△pod^* mice at the maturing stage (arrows in *Tjp1^△pod^*). Scale bars, 10 µm in the top panels and 2 µm in the bottom panels. (G) Transmission electron micrographs of P0 mouse podocytes demonstrating the presence of slit diaphragms at the maturing stage in the control mice (arrows in the top panel), but not in the *Tjp1^△pod^* mice (arrows in the bottom panel). Scale bars, 0.2 µm. (H) Albumin and higher molecular weight proteins were detected in urine of P0 *Tjp1^△pod^* mice.

To examine whether the expression of Tjp1affected the stability of nephrin and/or podocin, we established a stable cell line that expressed both proteins with or without Tjp1. The expression and the complex formation of the transfected molecules were confirmed by Western blotting and immunoprecipitation assay (Fig. 4A). Then we inhibited de novo protein synthesis in the transfectants using cycloheximide treatment (Fig. 4B). The expression of nephrin was reduced to almost half of the previous amount after 8 h, while a 50% reduction in podocin levels was observed after 2–4 h in the absence of Tjp1. When Tjp1 was coexpressed, the reduction of podocin was attenuated, while the expression of nephrin did not change significantly (Fig. 4B and C). Therefore, the podocin protein appeared to be stabilized by forming complex with Tjp1.

We also explored a possible mechanism for the increase of Synpo expression in the *Tjp1^△pod^* mice. The increase of Synpo expression was detected at 2 weeks of age (Fig. 3A); rather later than the decrease of podocin or nephrin (Fig. 3D), and almost at the same time when foot processes were found to be spread out (Fig. 2B). Since the Synpo expression was not significantly increased at 1 week of age when the foot process adhesion to the GBM was intact but the interdigitation was altered (Fig. 2C), we speculated that the alteration of foot process adhesion to the GBM might affect the expression of Synpo. To address this possibility, the mouse podocytes were seeded on type-I collagen-coated or uncoated plates and cultured for ∼16 h, and the Synpo expression in the podocytes was compared by Western blotting (Fig. 4D). We found that the amount of Synpo protein from the cells cultured on uncoated plates was greater than the amount from the coated plates, suggesting the possibility that the decreased adhesion to the basement membrane could upregulate the expression of Synpo protein in podocytes.

### *Tjp1* is potentially indispensable for the integration of slit diaphragm components in developing podocytes

It has been demonstrated that there are different developmental stages of the glomerulus present in the newborn mouse kidney [26]. At the comma-shaped body stage, the immature podocytes differentiate from the columnar epithelial cells which are connected by tight and adherens junctions. Those junctions migrate toward the basal domains during the S-shaped body stage. In the meantime, podocyte-specific molecules such as nephrin and podocin become to be expressed in the late S-shaped body stage. Subsequently, the cells begin to form broad foot processes during the capillary loop stage, and the tight and adherens junctions disappear and are replaced by the slit diaphragms during the maturing stage [11]. To assess whether *Tjp1* was implicated in the generation of slit diaphragms, we investigated the distribution of podocin and the structure of foot processes in developing podocytes in the newborn mouse kidney.

Before the maturing stage, in which the podocyte foot processes were not sufficiently differentiated to form slit diaphragms yet [11], [26], podocin was detected around nucleus (Fig. 4E, arrowheads) and basolateral membrane domains with a dot-like pattern (Fig. 4E, arrows) both in the control and *Tjp1^△pod^* mice in a similar manner. At the maturing stage, when slit diaphragms are formed between interdigitating foot processes, the linear staining pattern of podocin was observed in the control mice (Fig. 4F, arrows in control). On the other hand, podocin did not exhibit the organized linear distribution pattern in *Tjp1^△pod^* mice. Instead, podocin was observed as discontinuous dots (Fig. 4F, arrows in *Tjp1^△pod^*) as that in the 1-week-old or 2-week-old *Tjp1^△pod^* mice (Fig. 2D and Fig. S4A).

In several previous studies, the direct implication of the podocyte component in the generation of slit diaphragms was assessed by examining the ultrastructure of developing podocytes in newborn mutant mice that were missing the podocyte component [32], [33], [34]. According to those studies, we analyzed podocyte ultrastructure in P0 control and *Tjp1^△pod^* mice. Transmission electron microscopy revealed the presence of regularly spaced foot processes and slit diaphragms in maturing podocytes in P0 control mice (Fig. 4G, top panel). In contrast, foot processes were located close together and normal slit diaphragms were not observed in maturing podocytes in P0 *Tjp1^△pod^* mice (Fig. 4G, bottom panel). In addition, albumin was detected in the urine from the P0 *Tjp1^△pod^* mice (Fig. 4H). From these data, we assumed that Tjp1 could play a role in the generation of normal slit diaphragms for the blood filtration.

## Discussion

In this study, we demonstrated that the podocyte-specific deletion of *Tjp1*, a component of tight junctions in epithelial cells, impaired the formation and maintenance of blood filtration apparatus. *Tjp1^△pod^* mice exhibited extensive non-selective proteinuria. In addition to albumin, proteins larger than albumin were seen in the urine of *Tjp1^△pod^* mice not only at 6 weeks of age, but also at 2 weeks of age and P0. And we found more than 90% of glomeruli in the 6-week-old *Tjp1^△pod^* mice showed sclerosis. These data suggest that the loss of Tjp1 in podocytes results in persistent proteinuria and disturbance of renal function with the reduction of glomerular filtration ability and lead to growth retardation.

In the *Tjp1^△pod^* mice, the loss of slit diaphragms and foot process effacement appear to precede disorganization of the basement membrane from the observations that the GBM is mostly well-organized with the clear three layers at 2 weeks of age (Fig. S3E), while the organization of GBM is severely impaired at 6 weeks of age (Fig. 1G). In addition, the enlarged renal pelvis and atrophic renal papilla were observed in the *Tjp1^△pod^* mice at 6 weeks of age (Fig. 1E), but not at 2 weeks of age, indicating that this alteration was secondary consequence of profound damage of the glomerulus. Since the peritubular capillary stems from the efferent arterioles, diffuse global glomerulosclerosis is thought to causes tubular hypoxia, especially in the papilla, which is most vulnerable. Severe nephrotic syndrome also decreases circulating plasma volume, which also causes hypoperfusion and hypoxia of the kidney. The development of hypoperfusion in the *Tjp1^△pod^* mice is supported by pale appearance of the *Tjp1^△pod^* kidney (Fig. 1D). We therefore speculate that hypoxia is a potential mechanism underlining the papillary atrophy.

At the molecular level, the deletion of *Tjp1* in podocytes prevented the proper spatial arrangement of podocin at the prospective slit diaphragm region in the maturing stage (Fig. 4F). In addition, the stability of podocin, which has a short half-life, was decreased (Fig. 4B and C). Taking into consideration the lack of well-developed foot process interdigitation in the SEM image of the glomeruli of *Tjp1^△pod^* mice at 1 week of age (Fig. 2C), we speculate that these molecular alterations could be associated with the impairment of normal interdigitated architecture of foot processes, and lead to the disturbance of the foot process adhesion to the GBM as observed in the *Tjp1^△pod^* mice at 2 week of age (Fig. 2B), possibly because of the inability to handle the filtration pressure. In older mice, the foot processes remained disorganized but did not completely retract or disappear (Fig. 2A). This type of structural alteration may be similar to some models of glomerular disease that exhibit foot process flattening, rather than the complete loss *in vivo*, which is correlated with an increased podocyte spreading *in vitro* [35], [36].

The previous studies have shown that P-cadherin and β-catenin, both of which are adherens junction components, were not necessary for the development and maintenance of the slit diaphragm [37], [38]. Therefore, slit diaphragms could be characterized as modified tight junctions rather than adherens junctions. We speculate that for the generation of the functional slit diaphragms, the integration of two independent units, the pre-existing epithelial junction components and the newly synthesized podocyte-specific components, would be required at the final step of podocyte differentiation, in which Tjp1 is implicated. This hypothesis needs to be clarified by further studies.

We had previously demonstrated that the constitutive inactivation of *Tjp1* in mice resulted in an early embryonic lethality [39]; thus, it was unlikely that *Tjp1* was actually mutated in congenital human glomerular diseases. Nonetheless, considering the previous reports that *Tjp1* expression was decreased in glomerular diseases in human and animal models and our findings in the current study, the suppression of *Tjp1* could directly aggravate human glomerular disorders, which highlighted *Tjp1* as a potential therapeutic target.

## Materials and Methods

### Ethics statement

The care and use of all mice in this study were in accordance with the Guidelines for Proper Conduct of Animal Experiment (Science Council of Japan). All animal protocol was approved by the committee of the Care and Use of Laboratory Animals in Dokkyo Medical University (Permit Number: 06-517) and experimental work was performed in accordance with the ARRIVE guidelines [40]. All efforts were made to minimize suffering including housing mice in a specific pathogen-free unit in which the light cycle was maintained at 12 h light/12 h dark and room temperature was 21±2°C. No more than 5 mice were housed in one cage and given food and water ad libitum. Mice were sacrificed by terminal anaesthesia with isoflurane followed by cervical dislocation.

### Generation of *Tjp1^flox/flox^* mice and podocyte-specific *Tjp1* knockout mice

The targeting vector was constructed as follows: neomycin and puromycin selection markers flanked by FRT (NeoR) and F3 (PuroR), respectively, were inserted into intron 3 and intron 4, respectively, and flanked by loxP sites. This allows Flp-mediated removal of the selection markers and Cre-mediated deletion of *Tjp1* exon 4. The C57BL/6N ES cell line was grown on a mitotically inactivated feeder layer comprised of mouse embryonic fibroblasts (MEF) in DMEM High glucose medium containing 20% FBS (PAN) and 1200 u/mL Leukemia Inhibitory Factor (Millipore ESG 1107). 1×10^7^ cells and 30 µg of linearized DNA vector were electroporated (Biorad Gene Pulser) at 240 V and 500 µF. Puromycin selection (1 µg/mL) and G418 selection (200 µg/mL) started on day 2. Counterselection with Gancyclovir (2 µM) started on day 5 after electroporation. ES clones were isolated on day 8 and analyzed by Southern Blotting according to standard procedures after expansion and freezing of clones in liquid nitrogen. Blastocysts were microinjected with the targeted ES cells, transferred into the uterine horn of pseudo pregnant Balb/c females and chimeras were identified by coat color contribution. Highly chimeric males were bred to a C57BL/6 Flp-deleter strain and germ line transmission was identified by the presence of black, strain C57BL/6, offspring. *Tjp1^flox/flox^* mice were identified by genotyping PCR using primer 1 (5′-CTT CTC TGA CCC TAC ACA GCT ACC-3′) and primer 2 (5′-ATC GTG TGG GAA AGA CAA GC-3′) to obtain 279 bp and 471 bp fragments for the wild-type and conditional mutant alleles, respectively. To generate the podocyte-specific *Tjp1* knockout mice, *Tjp1^flox/flox^* mice were crossed with *Nphs1-Cre* transgenic mice, which drive Cre recombinase expression in podocytes under nephrin promoter [23], resulting in the generation of *Nphs1-Cre:Tjp1^flox/+^* mice. The *Tjp1^△pod^* (*Nphs1-Cre:Tjp1^flox/flox^*) mice were generated by crossing *Tjp1^flox/flox^* mice with *Nphs1-Cre:Tjp1^flox/+^* mice. All animal studies were approved by the committee on research animal care in our university.

### Histology and electron microscopy

The kidney samples were fixed with 4% paraformaldehyde and 2.5% glutaraldehyde in 0.1 M phosphate buffer (pH 7.4) overnight at 4°C. For the histology, the fixed kidneys were embedded in paraffin and sectioned at 2-μm thickness. The sections were processed for hematoxylin and eosin, periodic acid-Schiff (PAS), Jones' silver, or Masson's Trichrome staining. For the transmission electron microscopy, the kidneys were post-fixed with 1% OsO_4_, dehydrated, and embedded in epoxy resin. Ultrathin sections were prepared and stained with uranyl acetate and lead citrate. For the scanning electron microscopy, the kidneys were cut in 1-mm thick-sections, post-fixed with 1% OsO4, cryoprotected with 30% sucrose, and freeze–thawed with liquid nitrogen. Then, the blocks were dehydrated, frozen in t-butyl alcohol, and sublimated.

### Immunostaining and immunogold labeling

The cryosections were prepared from frozen kidney tissue samples and fixed with 2% PFA for 15 min at room temperature or with cold methanol for 10 min at −20 °C. After washing with phosphate buffered saline (PBS), the sections were blocked with 1% bovine serum albumin (BSA) in PBS and stained with the primary antibodies. Alexa-488- or Alexa-594-conjugated fluorophores (Invitorgen) were used in a final dilution of 1∶300 as secondary antibodies. For immunogold labeling, the kidneys were cut on a microtome at 50-μm thickness, cryoprotected, and freeze–thawed with liquid nitrogen. The sections were incubated with rabbit anti-podocin pAb (1∶100 dilution) and gold-conjugated anti-rabbit antibody, subsequently post-fixed with 1% OsO4, and dehydrated. After embedding in epoxy resin, ultrathin sections were prepared.

### Antibodies

The following primary antibodies were used: rat anti-Tjp1 (Santa Cruz Biotech), rabbit anti-Tjp1 (Invitrogen), rabbit anti-Tjp2 (Invitrogen), rabbit anti-Tjp3 (Invitrogen), rabbit anti-WT1 (Santa Cruz Biotech), goat anti-VE-cadherin (Santa Cruz Biotech), rabbit atni-Caludin2 (Invitrogen), guinea pig anti-Nephrin (Progen), rabbit anti-Podocin (Sigma-Aldrich), rabbit anti-Fyn (Sigma-Aldrich), rabbit anti-Synaptopodin (Sigma-Aldrich), rabbit anti-α-actinin-4 (Millipore), rabbit anti-CD2AP (Cell signaling), and rabbit anti-β-actin (Sigma-Aldrich).

### Assessment of proteinuria

Mouse urine (2 µL) was subjected to sodium dodecyl sulfate (SDS) polyacryl amide gel electrophoresis (SDS-APGE) followed by Coomassie brilliant blue staining. Colorimetric analysis of urinary protein was performed using a BCA Protein Assay Kit (Bio-Rad).

### Analyses of expression levels of the podocyte proteins in the glomerulus

Mouse glomerular fractions were isolated from the renal cortex using nylon mesh with the standard sieving method and homogenized in lysis buffer (1% SDS, 0.5 mM phenylmethylsulfonyl fluoride, 5 µg/mL leupeptin, 5 µg/mL antipain, 5 µg/mL chymostatin, 20 mM Tris-HCl pH 8.0). For mice that were younger than 1 week of age, protein lysates were extracted from the whole kidney. Protein concentrations of the supernatants after centrifugation (15,000 rpm × 10 min) were determined by detergent-compatible protein quantification assays (Bio-Rad); equal amounts of protein were used for the SDS-PAGE, which was followed by Western blotting analyses. After incubation with the primary antibodies, horseradish peroxidase (HRP)-conjugated secondary antibodies (GE Healthcare) and chemiluminescent substrate (Bio-Rad) were applied.

### Quantitative RT-PCR

RNA was isolated using TRIZOL (Invitrogen) and then reverse-transcribed with a PrimeScript RT reagent kit (Takara Bio.). Quantitative PCR was performed using a Real-Time PCR System 7300 (Applied Biosystems) and SYBR green master mix (Roche). All samples were normalized by the GAPDH expression using delta–delta Ct method. The following primer pairs were used: Nephrin 5′-AGGGTCGGAGGAGGATCGAA-3′ and 5′-GGGAAGCTGGGGACTGAAGT-3′; Podocin 5′-ACAAGGTTGATCTCCGTCTCCAG-3′ and 5′-TTTCCATGCGGTAGTAGCAGACAG-3′; CD2AP 5′-CAAGATGCCTGGAAGACGA-3′ and 5′-GCACTTGAAGGTGTTGAAAGAG -3′; Fyn 5′-TGCTGCCGCCTAGTAGTTCCC-3′ and 5′-CTCAGACACGACCGCGTAGAGC-3′; Synaptopodin 5′-CATCGGACCTTCTTCCTGTG-3′ and 5′-TCGGAGTCTGTGGGTGAG-3′; α-actinin-4 5′-TCCAGGACATCTCTGTGGAAG-3′ and 5′-CATTGTTTAGGTTGGTGACTGG-3′.

### Cell culture, transfection, and biochemical assays

Mouse fibroblast L-cells, in which endogenous Tjp1 was expressed at a very low level, were maintained on Dulbecco's Modified Eagle Medium supplemented with 10% fetal bovine serum (FBS) and transfected with pCAG-IRES-GFP-Nephrin, pCAG-Puro-Podocin, and pCAG-Neo-Tjp1 or pCAG-Neo control vector. Transfected cells were cultured in the presence of 500 µg/mL neomycin and 10 µg/mL puromycin for 14 days; cells positive for green fluorescent protein were collected using a cell sorter (BD). The expression of the transfected genes was confirmed by Western blotting. For immunoprecipitation, cell lysates were precleared with protein G-Sepharose 4FF beads (GE Healthcare) and incubated with the primary antibody or control IgG for 1 hr. Protein G-Sepharose 4FF beads were added to capture the immuno-complex, then washed with buffer A (20 mM Tris-HCl, 150 mM NaCl, 1 mM EDTA, 10% Glycerol, 1% NP-40, 1 mM PMSF, pH 7.4), followed by elution with Laemmli sample buffer (2% SDS, 10% Glycerol, 5% 2-mercaptoehtanol, 60 mM Tris-HCl pH 6.8). For the protein stability assay, the transfected cells were treated with 10 µM cycloheximide (Wako Chemicals) and lysed with Laemmli sample buffer at the indicated times. The mouse podocyte cell line was a kind gift from P. Mundel and K. Asanuma, and cultured as described previously [41]. The differentiated cells were seeded onto type-I collagen dishes or non-coated petri dishes and cultured for 16 h, then lysed with Laemmli sample buffer. The cell lysates were processed for SDS-PAGE, which was followed by Western blotting analyses.

### Statistical Analyses

Data were expressed as the mean ± SEM. Statistical significance was determined with the Student *t* test or ANOVA.

## Supporting Information

Figure S1 **Generation of** ***Tjp1*** **-floxed mice.** (A) Targeting strategy using the Cre/loxP system. Schematic of the mouse genomic locus for *Tjp1* (ZO-1 gene) is shown. The targeting vector introduces loxP-FRT-flanked Neo and F3-loxP-flanked Puro resistance cassettes 5′ and 3′, respectively, of *Tjp1* exon 4. After Flp recombination, the conditional allele contains a loxP-flanked exon 4 with residual 5′ FRT and 3′ F3 sites. Cre-recombination deletes the floxed exon 4, yielding the constitutive KO allele. Genotyping PCR primers to identify wild-type, *Tjp1*-floxed hetero (*Tjp1^flox/+^*) and homozygous (*Tjp1^flox/flox^*) animals are indicated with orange bars. The drawing is not to scale. (B) Confirmation of homologous recombination at the 5′- and 3′-sites. Schematic representation of the wild-type and targeted allele. The exon (Ex)-intron organization is shown with the selection casettes (Neo, Puro), the floxed arm of homology (FA), the short and long arms of homology (SA and LA, respectively), the 5′- and 3′-end probes (5e1 and 3e1, respectively) and the XcmI and BclII restriction enzyme cutting sites. Genomic DNA from the indicated ES cell clones were digested with XcmI and probed with 5e1 by Southern blot, showing an expected 10 kb band indicative homologous recombination at the 5′-end (top panels). Digestion with BclII followed by Southern blot hybridization with probe 3e1 yields an expected 17.4 kb band showing homologous recombination at the 3′-end (bottom panels). (C) Confirmation of single site integration. To confirm that homologous recombination only occurred at the *Tjp1* locus, genomic DNA was digested with PacI and AvrII and probed with a puromycin specific probe (puro). A single band of the expected 13.8 kb was obtained, consistent with a single site integration at the *Tjp1* locus. (D) Genotyping by PCR. Agarose gel electrophoresis of PCR products from wild-type, *Tjp1^flox/+^* and *Tjp1^flox/flox^* mice using the primers indicated in panel A. Fragments of 279 bp and 471 bp are indicative of the wild-type and floxed allele, respectively. WT, wild-type.
(PDF)

Figure S2 **Immunostaining of Tjp1 and cell-type specific markers in the glomerulus.** (A–C) Localization of Tjp1 in the glomerulus was examined by co-staining with cell-type specific markers. Frozen kidney sections of the control and *Tjp1^△pod^* mice were stained using antibodies against Tjp1 with either podocyte marker WT1 (A), endothelial cell marker VE-cadherin (B), or Bowman's capsule epithelial cell marker Claudin-2 (C). In control mice, Tjp1 was detected in podocytes, endothelial cells, and Bowman's capsule epithelial cells. In the *Tjp1^△pod^* mice, Tjp1 was still expressed in endothelial cells and Bowman's capsule epithelial cells as in control mice, but was absent from WT1-positive podocytes. See also Fig. S4A which showed co-staining images of Tjp1 and the podocyte slit diaphragm protein podocin. Scale bars, 10 µm. (D) The glomerular lysates obtained from the control and *Tjp1^△pod^* mice were processed for Western blotting analysis. The reduction of Tjp1 protein was observed in the glomerulus of *Tjp1^△pod^* mice.
(PDF)

Figure S3 **Tjp1, but not Tjp2 or Tjp3, plays crucial role for the establishment of the slit diaphragm.** (A and B) Kidney sections from the control and *Tjp1^△pod^* mice were stained with antibodies against Tjp2 (A) and Tjp3 (B). The expression and localization of either protein was not affected by the podocyte-specific deletion of *Tjp1*. Scale bars, 10 µm. (C and D) Histological analyses of kidneys from the control and *Tjp1^△pod^* mice at 2 weeks and 4 weeks of age demonstrated a progressive disorder. The glomerulus was severely impaired and the dilated renal tubules were filled with protein casts in the *Tjp1^△pod^* mice at 4 weeks of age (C). *Tjp1^△pod^* mice at 2 weeks of age exhibited milder but obvious defects in the glomerulus (D). Scale bars, 20 µm. (E) Global foot process effacement was observed in the *Tjp1^△pod^* mice at 2 weeks of age. The slit diaphragm was absent and aberrant contacts between the foot processes were detected (bottom panels). Scale bars, 2 µm (top panels), 0.4 µm (bottom panels). (F) *Tjp1^△pod^* mice at 2 weeks of age exhibited significant proteinuria.
(PDF)

Figure S4 **A close inspection of podocin and synaptopodin (Synpo) localization.** (A) The higher magnification immunostaining images of Tjp1 and podocin in the control and *Tjp1^△pod^* mice at 2 weeks of age. Scale bars, 2.5 µm. (B) The precise localization of podocin was determined by immunoelectron microscopy. Podocin was specifically detected at the slit diaphragm in the control mice. On the other hand, podocin labeling was observed on the cytoskeletal filaments and the collapsed structure near the GBM in the *Tjp1^△pod^* mice. Scale bars, 0.4 µm. (C) The higher magnification images of Synpo distribution in the control and *Tjp1^△pod^* mice at 2 weeks of age. Scale bars, 2.5 µm.
(PDF)
