# Supplementary figures and images for: The Structural and Functional Organization of the Podocyte Filtration Slits Is Regulated by Tjp1/ZO-1
Source: PLoS One. 2014 Sep 3;9(9):e106621. doi: 10.1371/journal.pone.0106621 (PMC4153657; doi:10.1371/journal.pone.0106621)

# Figure S1

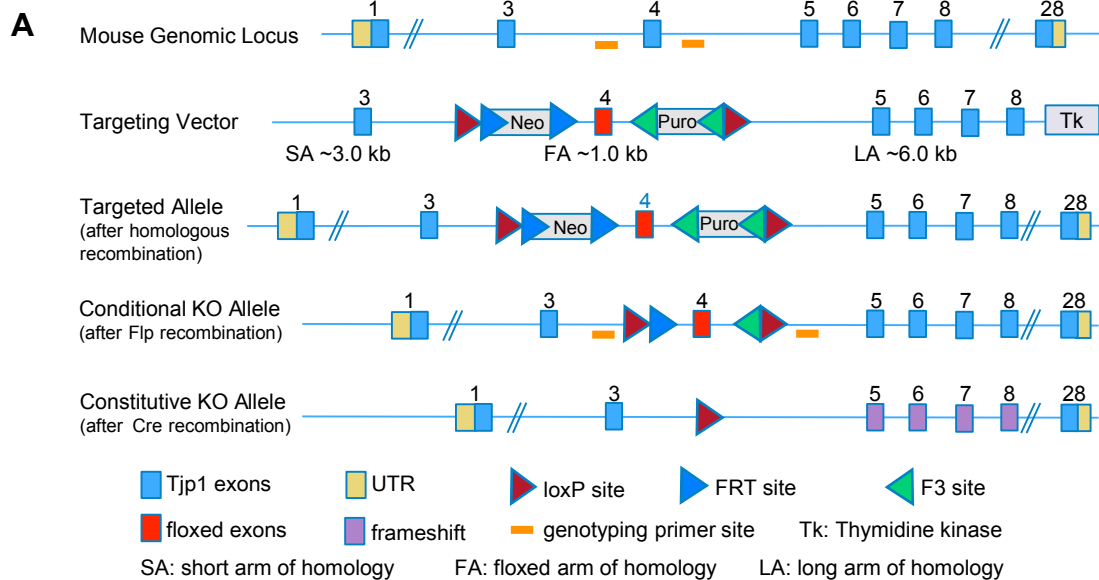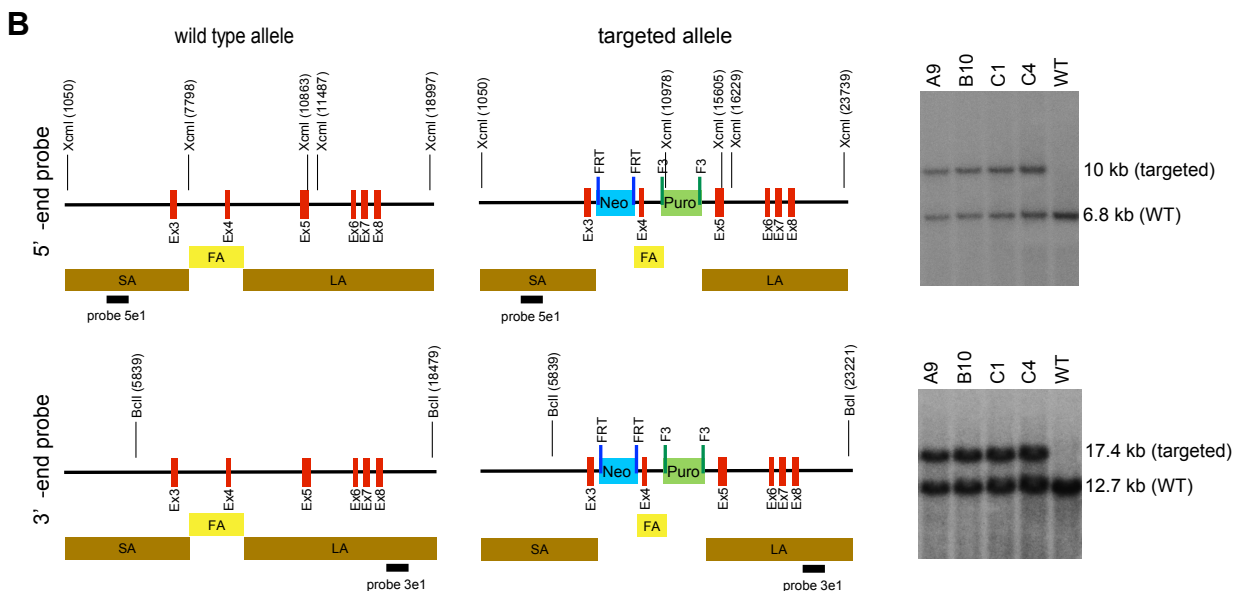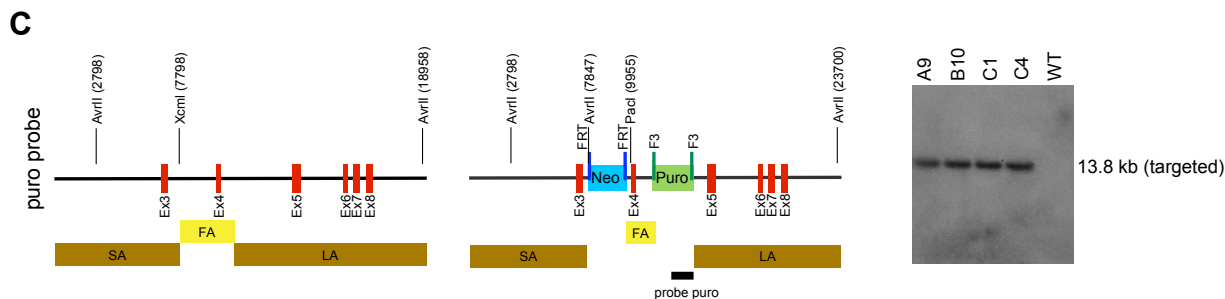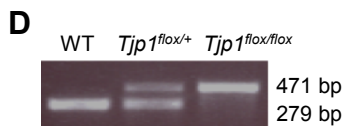

Supplement: Figure S1 — Generation of Tjp1 -floxed mice. (A) Targeting strategy using the Cre/loxP system. Schematic of the mouse genomic locus for Tjp1 (ZO-1 gene) is shown. The targeting vector introduces loxP-FRT-flanked Neo and F3-loxP-flanked Puro resistance cassettes 5′ and 3′, respectively, of Tjp1 exon 4. After Flp recombination, the conditional allele contains a loxP-flanked exon 4 with residual 5′ FRT and 3′ F3 sites. Cre-recombination deletes the floxed exon 4, yielding the constitutive KO allele. Genotyping PCR primers to identify wild-type, Tjp1-floxed hetero (Tjp1flox/+) and homozygous (Tjp1flox/flox) animals are indicated with orange bars. The drawing is not to scale. (B) Confirmation of homologous recombination at the 5′- and 3′-sites. Schematic representation of the wild-type and targeted allele. The exon (Ex)-intron organization is shown with the selection casettes (Neo, Puro), the floxed arm of homology (FA), the short and long arms of homology (SA and LA, respectively), the 5′- and 3′-end probes (5e1 and 3e1, respectively) and the XcmI and BclII restriction enzyme cutting sites. Genomic DNA from the indicated ES cell clones were digested with XcmI and probed with 5e1 by Southern blot, showing an expected 10 kb band indicative homologous recombination at the 5′-end (top panels). Digestion with BclII followed by Southern blot hybridization with probe 3e1 yields an expected 17.4 kb band showing homologous recombination at the 3′-end (bottom panels). (C) Confirmation of single site integration. To confirm that homologous recombination only occurred at the Tjp1 locus, genomic DNA was digested with PacI and AvrII and probed with a puromycin specific probe (puro). A single band of the expected 13.8 kb was obtained, consistent with a single site integration at the Tjp1 locus. (D) Genotyping by PCR. Agarose gel electrophoresis of PCR products from wild-type, Tjp1flox/+ and Tjp1flox/flox mice using the primers indicated in panel A. Fragments of 279 bp and 471 bp are indicative of [file pone.0106621.s001.pdf]

**Figure S2**

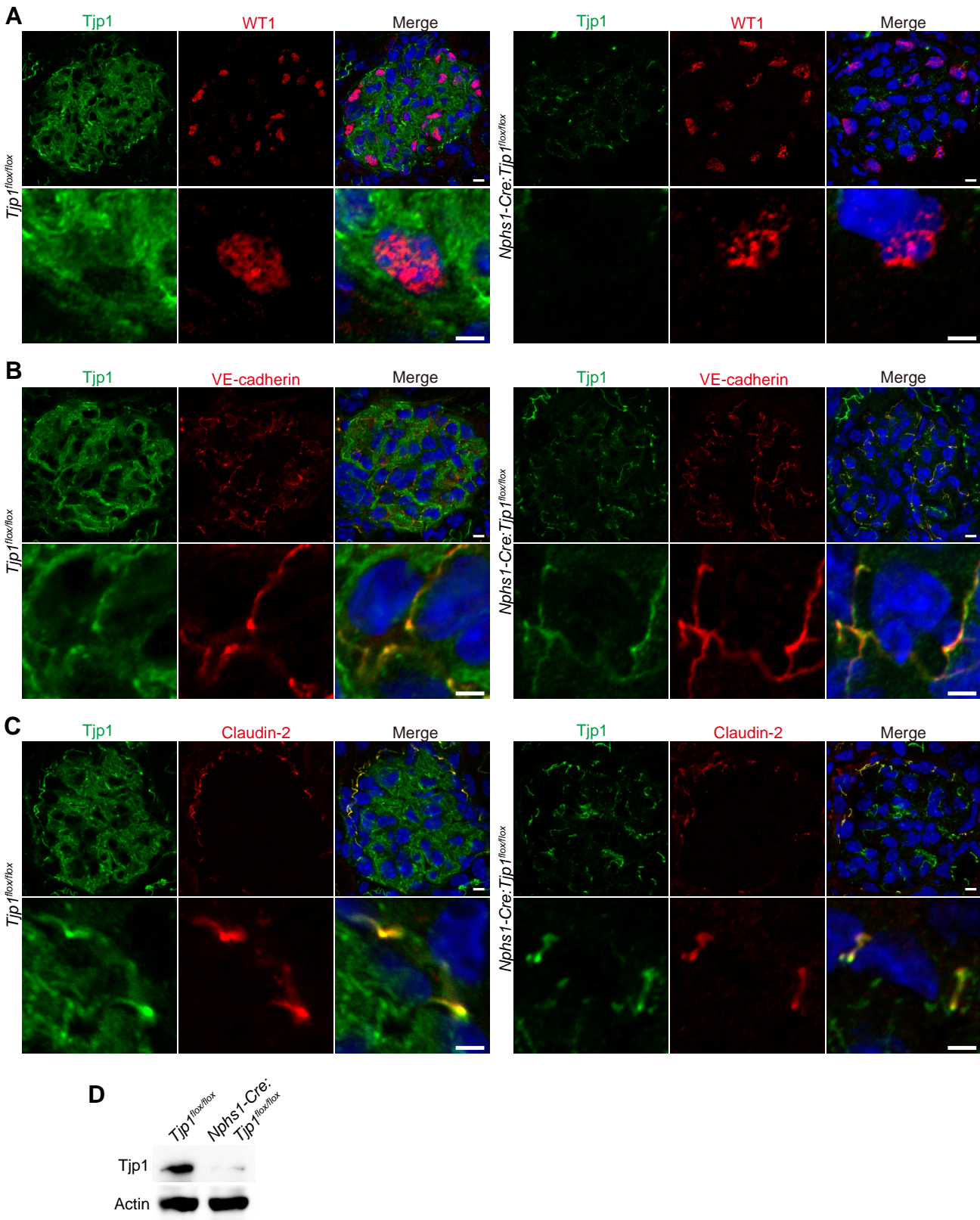

Supplement: Figure S2 — Immunostaining of Tjp1 and cell-type specific markers in the glomerulus. (A–C) Localization of Tjp1 in the glomerulus was examined by co-staining with cell-type specific markers. Frozen kidney sections of the control and Tjp1△pod mice were stained using antibodies against Tjp1 with either podocyte marker WT1 (A), endothelial cell marker VE-cadherin (B), or Bowman's capsule epithelial cell marker Claudin-2 (C). In control mice, Tjp1 was detected in podocytes, endothelial cells, and Bowman's capsule epithelial cells. In the Tjp1△pod mice, Tjp1 was still expressed in endothelial cells and Bowman's capsule epithelial cells as in control mice, but was absent from WT1-positive podocytes. See also Fig. S4A which showed co-staining images of Tjp1 and the podocyte slit diaphragm protein podocin. Scale bars, 10 µm. (D) The glomerular lysates obtained from the control and Tjp1△pod mice were processed for Western blotting analysis. The reduction of Tjp1 protein was observed in the glomerulus of Tjp1△pod mice. (PDF) [file pone.0106621.s002.pdf]

**Figure S3**

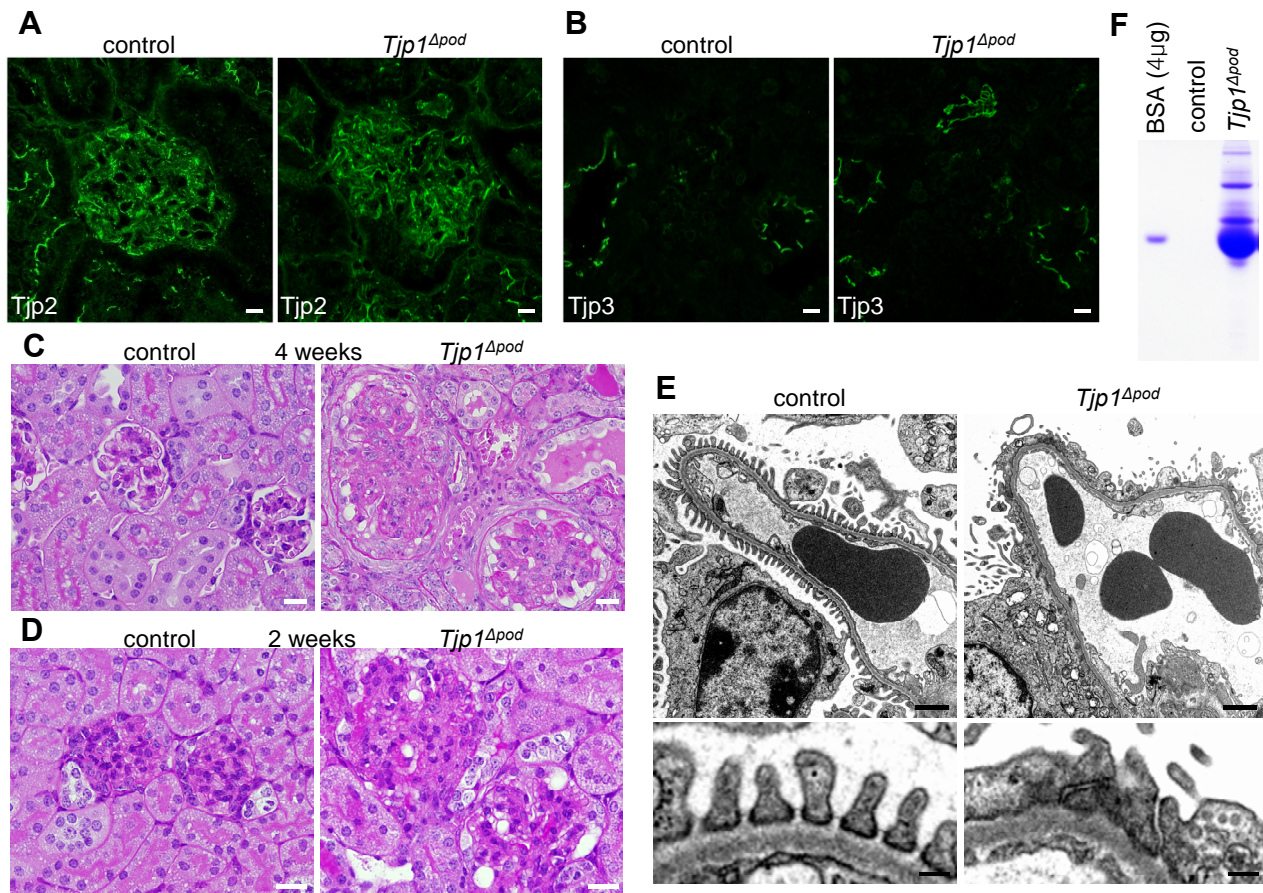

Supplement: Figure S3 — Tjp1, but not Tjp2 or Tjp3, plays crucial role for the establishment of the slit diaphragm. (A and B) Kidney sections from the control and Tjp1△pod mice were stained with antibodies against Tjp2 (A) and Tjp3 (B). The expression and localization of either protein was not affected by the podocyte-specific deletion of Tjp1. Scale bars, 10 µm. (C and D) Histological analyses of kidneys from the control and Tjp1△pod mice at 2 weeks and 4 weeks of age demonstrated a progressive disorder. The glomerulus was severely impaired and the dilated renal tubules were filled with protein casts in the Tjp1△pod mice at 4 weeks of age (C). Tjp1△pod mice at 2 weeks of age exhibited milder but obvious defects in the glomerulus (D). Scale bars, 20 µm. (E) Global foot process effacement was observed in the Tjp1△pod mice at 2 weeks of age. The slit diaphragm was absent and aberrant contacts between the foot processes were detected (bottom panels). Scale bars, 2 µm (top panels), 0.4 µm (bottom panels). (F) Tjp1△pod mice at 2 weeks of age exhibited significant proteinuria. (PDF) [file pone.0106621.s003.pdf]
