# Supplementary material for: The Structural and Functional Organization of the Podocyte Filtration Slits Is Regulated by Tjp1/ZO-1
Source: PLoS One. 2014 Sep 3;9(9):e106621. doi: 10.1371/journal.pone.0106621 (PMC4153657; doi:10.1371/journal.pone.0106621)
Supplement: Figure S4 — A close inspection of podocin and synaptopodin (Synpo) localization. (A) The higher magnification immunostaining images of Tjp1 and podocin in the control and Tjp1△pod mice at 2 weeks of age. Scale bars, 2.5 µm. (B) The precise localization of podocin was determined by immunoelectron microscopy. Podocin was specifically detected at the slit diaphragm in the control mice. On the other hand, podocin labeling was observed on the cytoskeletal filaments and the collapsed structure near the GBM in the Tjp1△pod mice. Scale bars, 0.4 µm. (C) The higher magnification images of Synpo distribution in the control and Tjp1△pod mice at 2 weeks of age. Scale bars, 2.5 µm. (PDF) [file pone.0106621.s004.pdf]

Figure S4

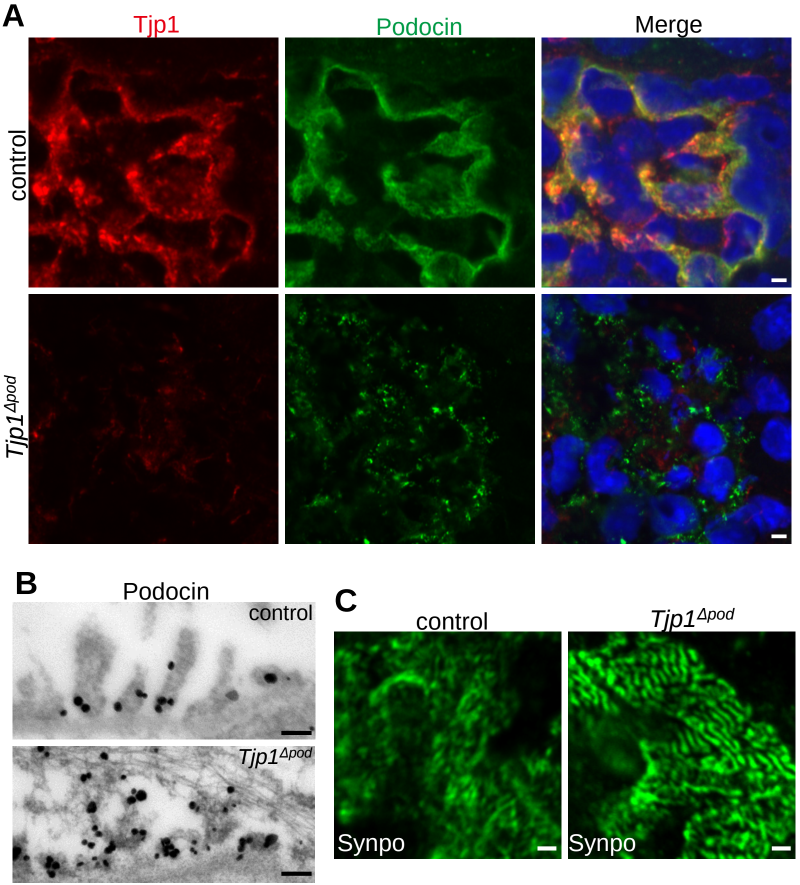
A
Tjp1
Podocin
Merge
control
Tjp1^Δpod
B
Podocin
control
Tjp1^Δpod
C
control
Tjp1^Δpod
Synpo
Synpo
